# Career Calling and Professional Match Among Chinese Graduates: The Roles of Career Loyalty and Industry Income

**DOI:** 10.3390/bs15111472

**Published:** 2025-10-29

**Authors:** Ting Zhang, Huan Zhang, Guan Ren, Hongxi Ge, Ziqiang Zhang

**Affiliations:** 1 School of Government, Beijing Normal University, Beijing 100875, China; 202331240009@mail.bnu.edu.cn (T.Z.);; 2 Surrey Business School, University of Surrey, Surrey GU2 7XH, UK; zz01255@surrey.ac.uk

**Keywords:** Chinese graduates, career loyalty, career calling, professional match, industry income

## Abstract

This study investigates the role of career calling in shaping Chinese graduates’ professional match, with a focus on the mediating role of career loyalty and the moderating effect of industry income. Drawing on Conservation of Resources (COR) theory and person–environment (P–E) fit theory, we developed a three-wave, multi-source design with 2025 graduates across diverse industries. The results reveal that career calling significantly enhances professional match, and this relationship is fully mediated by career loyalty. Moreover, industry income strengthens the positive effect of calling, suggesting that external rewards amplify internal motivation in achieving sustainable career outcomes. Theoretically, the study extends calling research into the graduate labor market and integrates contextual economic factors into the COR and P–E fit frameworks. Practically, the findings highlight the importance of cultivating career calling through higher education, organizational practices, and policy initiatives to improve workforce alignment and long-term career sustainability.

## 1. Introduction

The transition from university to employment represents a pivotal stage in individuals’ career trajectories, yet mismatches between academic majors and occupational choices remain a widespread challenge across labor markets (Didier, 2024). For many graduates, securing employment that aligns with their field of study is not only an indicator of efficient human capital utilization but also a determinant of long-term career sustainability. In China, this issue has become increasingly salient as the rapid expansion of higher education has outpaced the labor market’s absorptive capacity, leading to intensified competition, structural unemployment, and frequent instances of major-occupation mismatch (Shi, 2024). Given the societal and personal costs of such mismatches—including reduced productivity, lower career satisfaction, and higher turnover—it is essential to understand the psychological and contextual mechanisms that shape graduates’ ability to translate educational investment into congruent occupational outcomes.

Among the many factors influencing career alignment, individuals’ intrinsic motivational orientations have attracted growing scholarly attention. Career calling, defined as a profound sense that one’s work is meaningful, socially valuable, and aligned with personal identity, has been shown to affect career decision-making, persistence, and adaptability (Dobrow & Tosti-Kharas, 2012). For young professionals, calling not only fuels persistence in pursuing careers consistent with their academic training but also enhances the willingness to overcome labor market barriers. However, while existing research highlights calling’s role in fostering positive work attitudes, few studies have directly examined its contribution to Major-Occupation Match, particularly in the context of emerging economies where educational expansion has dramatically reshaped labor dynamics.

In addition to personal orientations, organizational and environmental conditions also influence whether calling translates into concrete career outcomes. Career loyalty—conceptualized as a strong psychological attachment to one’s occupation—may serve as a critical mediating mechanism (Lestari et al., 2021). Graduates who perceive their career as a calling are likely to internalize stronger occupational loyalty, which in turn sustains their efforts to remain in and adapt to jobs aligned with their academic major. Moreover, economic structures, such as industry income levels, can amplify or constrain this process. Higher-paying industries not only signal stronger labor demand but also provide tangible resources that reinforce the alignment between calling and occupational choices. This perspective aligns with both Conservation of Resources (COR) theory, which emphasizes individuals’ motivation to acquire and preserve valuable resources (Olga & Nurraihan, 2025), and person–environment (P–E) fit theory, which highlights the congruence between individuals’ characteristics and external environments as a determinant of career outcomes (Kaur & Kang, 2021).

To address these gaps, this study investigates how career calling influences graduates’ Major-Occupation Match through the mediating mechanism of job loyalty and how industry income moderates this relationship. By integrating motivational, attitudinal, and contextual factors into one framework, we provide a more comprehensive understanding of how educational investment translates into effective career alignment.

**Figure 1:**
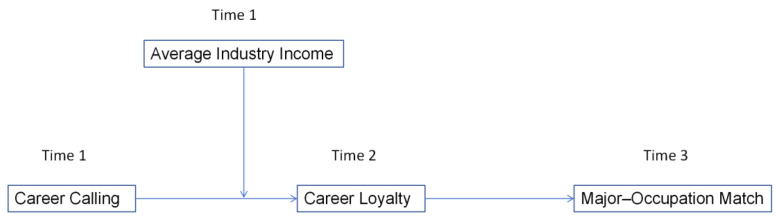
Theoretic Model.

Using a three-wave, multi-source, time-series cross-sectional design, we collected data from Chinese graduates and their supervisors across multiple provinces. Career calling was measured at Time 1, career loyalty at Time 2, and Major-Occupation Match at Time 3, with industry-level income data of 2024 drawn from official statistics (National Bureau of Statistics of China, 2025). This design enhances temporal validity and reduces concerns about common method bias by combining individual perceptions with objective economic indicators (Figure 1).

This study makes four major contributions to the literature. First, by focusing on the underexplored issue of Major-Occupation Match in the Chinese context, we provide culturally grounded insights into graduate employment dynamics. Second, we extend the literature on career calling by identifying its influence on structural career outcomes beyond subjective well-being. Third, we highlight the mediating role of career loyalty and the moderating role of industry income, thereby offering a nuanced model that integrates personal motivation with contextual factors. Fourth, our multi-stage, multi-source design advances methodological rigor by incorporating both longitudinal data and objective labor market statistics. Collectively, this study offers novel theoretical and practical implications for understanding how graduates’ internal resources and external conditions interact to shape their career alignment and long-term success.

## 2. Literature Review

The transition from higher education to employment is a pivotal stage in graduates’ career trajectories, and scholars have long emphasized the importance of achieving congruence between an individual’s academic specialization and occupational role (Etzel & Nagy, 2021). This congruence, commonly referred to as Major-Occupation Match (MOM), represents the alignment between the field of study and the professional domain in which graduates commence their careers. A substantial body of research indicates that MOM contributes positively to wages, job satisfaction, and early career development (Na et al., 2024). Conversely, Major-Occupation Mismatch—where graduates work in roles unrelated to their training—has been associated with underutilization of human capital, reduced job satisfaction, and diminished professional commitment (Romanò & Nazio, 2025). Given the expansion of higher education systems and the complexity of contemporary labor markets, especially in China, understanding the determinants of MOM is both theoretically significant and practically urgent.

One underexplored psychological factor that may shape graduates’ likelihood of achieving MOM is career calling. Defined as a profound sense of purpose and passion toward one’s work, career calling has been conceptualized as a motivational resource that orients individuals toward goal-consistent career pathways (Duffy et al., 2018). Beyond attitudinal outcomes such as life satisfaction or meaning in work, recent research highlights the role of calling in shaping behavioral outcomes, including persistence in one’s chosen field and adaptability during career transitions (Dobrow et al., 2023). This suggests that career calling may play a decisive role in whether graduates pursue occupations aligned with their academic specialization.

However, the relationship between calling and MOM is unlikely to be direct alone. It is important to consider career loyalty—conceptualized as occupational commitment—as a mediating mechanism that explains how calling translates into tangible career alignment (Setyaningrum et al., 2025). In addition, contextual moderators such as industry-level average income may amplify or attenuate these mechanisms, reflecting broader labor-market realities that either enable or constrain individuals’ pursuit of calling-consistent careers (Sam & Song, 2022).

The following review is structured to sequentially build the theoretical rationale and empirical evidence for four hypotheses. First, we examine the direct association between career calling and MOM (H1). Second, we introduce career loyalty as a mediator in this relationship (H2). Third, we theorize the moderating role of industry average income on the calling–loyalty link (H3). Finally, we integrate these into a moderated mediation framework in which industry income strengthens the indirect effect of calling on MOM via loyalty (H4).

### 2.1. Career Calling and Major-Occupation Match (H1)

The construct of career calling has evolved into a central theme in vocational psychology over the past two decades. Recent meta-analyses and empirical studies underscore its significance for individual well-being and career development (Mulyana et al., 2024). Duffy et al. (2018) describe calling as a “guiding compass” that influences career decisions and persistence, while more recent research (Mauno et al., 2025) finds that calling predicts proactive career behaviors and exploration among university students. These findings highlight that calling is not merely an attitudinal orientation but also translates into concrete decisions during school-to-work transitions.

Empirical evidence shows that individuals with higher levels of calling are more likely to persist in their chosen professional domains. For instance, Shen et al. (2021) found that students with stronger callings demonstrated greater career decision-making and lower turnover intentions within their fields of study. Similarly, researchers reported that calling predicted vocational identity clarity, which, in turn, shaped career choice consistency (Chen & Zhang, 2023). These studies collectively suggest that calling enhances psychological resources such as adaptability, identity clarity, and persistence—factors that increase the probability of graduates entering occupations congruent with their academic majors.

Person–environment (P–E) fit theory provides a theoretical foundation for linking calling to MOM. Calling often develops when individuals perceive their skills and values to be aligned with their occupational environments (Duffy et al., 2018). This alignment fosters a stronger intention to seek work in domains congruent with their education and personal values. Empirical research confirms that calling is associated with higher perceptions of person–job fit (Duffy et al., 2024). In turn, higher perceived fit predicts stronger intentions to work in related fields (Kaur & Kang, 2021). Thus, calling may indirectly shape MOM through enhancing perceived fit and guiding decision-making toward field-consistent roles.

Studies focusing specifically on transitions from education to work highlight the relevance of calling for MOM. Dobrow and Tosti-Kharas (2012), in a longitudinal study, found that students with higher levels of calling were more likely to secure employment aligned with their professional training after graduation. Similarly, a survey of Chinese undergraduates revealed that calling positively predicted persistence in licensure-required pathways (Jackson & Tomlinson, 2022). These findings underscore that calling plays a motivational role in achieving MOM.

Taken together, the evidence suggests that graduates who experience a strong sense of calling are more likely to pursue occupations congruent with their majors. Therefore, we propose:

**Hypothesis** **1.** *Graduate college students’ career calling is positively related to their Major-Occupation Match.*

### 2.2. Career Loyalty as a Mediator (H2)

While calling may exert a direct effect on MOM, it is important to acknowledge the underlying psychological mechanisms that transform motivational orientations into behavioral outcomes (Cheng et al., 2023). One such mechanism is career loyalty, which we conceptualize in line with the occupational commitment literature. Career loyalty reflects an individual’s identification with, attachment to, and willingness to invest effort in a specific professional domain (Olga & Nurraihan, 2025). It is distinguished from organizational commitment in that it transcends a specific employer and focuses on allegiance to the occupation itself.

Recent evidence suggests that career calling fosters stronger career loyalty. Researcher reported that individuals with a pronounced sense of calling exhibit heightened professional identity and are more committed to investing in domain-specific skill development (Verma et al., 2025). Likewise, Park and Kang (2020) found that calling among nursing students predicted higher occupational commitment, mediated through learning engagement. These findings indicate that calling motivates individuals to anchor their long-term career trajectories in a specific profession, thereby cultivating loyalty.

Theoretically, self-determination theory provides an explanatory lens: calling represents autonomous motivation, which fuels sustained engagement and commitment to chosen domains (Ryan & Deci, 2020). When individuals perceive their work as intrinsically meaningful and socially valuable, they are more likely to internalize professional values and maintain loyalty to their field.

Career loyalty, in turn, is a proximal predictor of MOM. Studies consistently demonstrate that higher occupational commitment predicts stronger intentions to remain in the profession and a lower likelihood of switching fields. For example, Low et al. (2022) showed that teachers with higher occupational commitment were more likely to stay in teaching despite external challenges. Similarly, occupational commitment among engineers has been linked to persistence in STEM careers (Khan et al., 2024). Translating these findings to graduates, career loyalty is expected to increase the likelihood of choosing initial jobs aligned with one’s major, as individuals are motivated to practice within their profession rather than pursuing unrelated opportunities.

The integration of these two processes—calling fostering loyalty, and loyalty predicting MOM—suggests a mediated relationship. That is, calling enhances career loyalty, which in turn promotes MOM. Empirical support for such mediated pathways has emerged in adjacent literatures: for example, Herrmann (2025) demonstrated that career adaptability mediated the relationship between calling and career persistence. Analogously, it is plausible that loyalty functions as the mediating mechanism through which calling translates into professional alignment.

Accordingly, we hypothesize:

**Hypothesis** **2.** *Graduate college students’ career calling positively affects their Major-Occupation Match through career loyalty.*

### 2.3. Industry Average Income as a Moderator (H3)

The role of industry-level average income provides an important contextual factor in understanding how career calling translates into loyalty. While calling represents an internalized sense of purpose, its translation into durable professional commitment may depend on external economic conditions. According to the Conservation of Resources (COR) theory, individuals strive to acquire, protect, and leverage resources, and the availability of contextual resources influences whether internal motivations can be effectively enacted (Bardoel & Drago, 2021). In labor markets characterized by high average industry income, graduates are likely to perceive stronger resource availability—higher salaries, better development opportunities, and greater social recognition—which can strengthen the conversion of calling into career loyalty.

Recent empirical studies confirm that wages differ significantly across industries, and such differences shape career choices and professional persistence. For example, researchers reported that graduates entering high-paying industries exhibited stronger intentions to remain in their occupational domain, largely due to reduced financial strain and higher return expectations (Gu & Zhu, 2023). Similarly, Lin and Xiong (2024) found that inter-industry wage disparities in China influenced students’ perceptions of occupational prestige and subsequent commitment to career pathways. These findings support the argument that industry income signals long-term career viability, thereby reinforcing professional loyalty among those with a strong calling.

Compensation has long been studied as a determinant of work attitudes, but recent research highlights its relevance for occupational-level commitment. Study demonstrated that pay satisfaction significantly predicted affective occupational commitment among healthcare professionals (Akinyemi et al., 2022). Likewise, other studies showed that salary levels moderated the relationship between intrinsic motivation and organizational commitment, suggesting that financial rewards amplify the expression of motivational orientations (Wang et al., 2022). Extending these insights, it is reasonable to expect that average industry income strengthens the link between calling and loyalty, as higher wages validate and reinforce the pursuit of one’s vocational passion.

Taken together, the theoretical reasoning and empirical findings suggest that the positive effect of career calling on career loyalty is contingent on industry average income. Therefore, we propose:

**Hypothesis** **3.** *The average industry income of graduates’ majors moderates the relationship between career calling and career loyalty, such that the relationship is stronger when average industry income is high and weaker when it is low.*

### 2.4. Moderated Mediation: Integrating Industry Income (H4)

Beyond its moderating role on the direct calling–loyalty relationship, industry average income may also condition the indirect effect of calling on MOM through loyalty. This conceptualization follows the moderated mediation framework, which posits that contextual variables can strengthen or weaken the magnitude of mediated pathways. Specifically, when average industry income is high, calling is more effectively transformed into loyalty (H3), and loyalty in turn predicts stronger MOM (H2). Consequently, the overall indirect effect of calling on MOM via loyalty should be amplified in high-income industries (Bhatti, 2025).

In the context of graduates’ transition to work, industry income serves as both an economic and symbolic resource. Economically, higher-paying industries reduce opportunity costs and enhance perceived returns on investment in one’s career. Symbolically, they convey social prestige and validation of one’s professional choice. When combined with a strong career calling, these signals foster stronger loyalty, which in turn raises the probability of achieving MOM. Conversely, in low-income industries, even individuals with a strong calling may find it difficult to sustain loyalty, thereby weakening the indirect effect on MOM.

Accordingly, we posit:

**Hypothesis** **4.** *The average industry income of graduates’ majors moderates the indirect effect of career calling on Major-Occupation Match through career loyalty, such that the indirect relationship is stronger under high average industry income.*

## 3. Method

### 3.1. Participants and Procedure

This study was approved by the Ethics Committee of the School of Social Development and Public Policy at Beijing Normal University (approval number: SSDPP-HSG 2023003). Following ethical approval, data collection was conducted in three waves between January 2023 and July 2025.

From January to December 2023, we recruited participants through an online platform. Specifically, invitations were distributed to graduating students in China via WeChat, a widely used social communication application that allows group-based messaging and information sharing. Participation was voluntary, and each participant received a 10 RMB reward upon completing the questionnaire, provided that their responses passed a quality check conducted by the research team. Eligible participants were Chinese university students expected to graduate in June 2025. To verify student status, participants were required to upload a photograph of their student ID card. To protect their privacy, they were permitted to obscure their names and ID numbers on the photograph. All participants provided informed consent and agreed to participate in a longitudinal study using multi-source, multi-wave data collection. By January 2024, a total of 10,000 students had been successfully recruited for the baseline survey (Time 1). However, in the subsequent stages, some participants discontinued their involvement simply because they became too busy with work or had other personal reasons for withdrawal.

At Time 1 (January–March 2024), we assessed participants’ career calling. In Time 2 (July–September 2024), we re-contacted the same sample to collect data on career loyalty. A total of 7354 students agreed to continue their participation at this stage. In China, university students typically go through two main recruitment periods: the fall recruitment season (September–December) and the spring recruitment season (March–June of the following year). Thus, we conducted the final follow-up at Time 3 (July 2025) to capture their employment outcomes. At this stage, 5321 students agreed to participate. Among them, 1422 had not secured employment, and 1874 had chosen to pursue further graduate education. Excluding these students, the final valid sample comprised 2025 employed graduates, whose data across all three waves were included in the present study. Data from students who did not participate in all three waves were excluded from the analyses.

Of the final sample, 1015 were male and 1010 were female, with an average age of 21.1 years (SD = 1.92). Regarding employment outcomes, 1122 graduates (55.4%) reported working in jobs related to their academic major, while 903 graduates (44.6%) reported working in jobs unrelated to their major.

### 3.2. Measures

Brislin’s method (Wu et al., 2023) was employed during the back-translation process in this study. A five-point Likert scale was used to assess the studied concepts, with responses ranging from 1 (“totally disagree”) to 5 (“totally agree”).

#### 3.2.1. Major-Occupation Match

Major-Occupation Match (MOM) was assessed using objective data. We compared each graduate’s reported field of study with the industry category of their current job. If the graduate’s job fell within the same industry category as their academic major, the variable was coded as 1 (matched); if not, it was coded as 0 (mismatched).

To ensure consistency and comparability, the classification of industry categories followed the official categories published in the 2024 National Bureau of Statistics (NBS) report on average industry wages in urban units (National Bureau of Statistics of China, 2025). This classification system distinguishes broad sectors of economic activity (e.g., manufacturing, education, financial industry) and has been widely used in national labor statistics. The full list of industry categories used in this study is presented in Table A1.

This operationalization of MOM is consistent with prior research adopting objective measures of horizontal mismatch, where the degree of alignment between the field of study and the occupational domain is treated as a binary indicator (e.g., matched vs. mismatched; Cheng et al., 2023; Salas-Velasco, 2021).

#### 3.2.2. Career Calling

Career calling was measured using the Career Calling Scale developed by C. Zhang et al. (2015). This scale was originally constructed based on a sample of Chinese college students, reflecting local cultural contexts and developmental characteristics. It consists of three dimensions—altruistic contribution, guiding force, and meaning and value—with a total of 11 items. A sample item is: “I want to be engaged in an occupation that is beneficial to others”. Item 3 is reverse-coded, and its score was reversed during data processing to ensure consistency across the measurement. All items were rated on a five-point Likert scale ranging from 1 (strongly disagree) to 5 (strongly agree). In the present study, the Cronbach’s alpha for this scale was 0.82, indicating high internal reliability.

#### 3.2.3. Career Loyalty

Career loyalty was measured using the scale developed by Yao and Wang (2008) and adapted from Ding et al. (2012), which includes seven items. A sample item is: “Working in a job related to my academic major is my best choice.” All items were rated on a five-point Likert scale ranging from 1 (strongly disagree) to 5 (strongly agree). In the present study, the Cronbach’s alpha for this scale was 0.86, indicating good internal consistency.

#### 3.2.4. Average Industry Income

Average industry income was measured using objective data from the National Bureau of Statistics of China (2025). Specifically, we used the 2024 Average Annual Wages of Employed Persons in Urban Units report, which provides official statistics on average annual wages across major industries in China and reports wage levels for the most recently completed full year. As such, the data provide an accurate reflection of the current conditions in the labor market. These industry-level wage data served as an external indicator of labor market resource availability for graduates’ academic majors.

To link participants’ majors with industry-level economic conditions, each graduate’s field of study was matched with the corresponding industry category as defined by the NBS classification system. The classification standard follows the categories published in the 2024 report, which cover a broad set of economic sectors (e.g., manufacturing, education, financial industry, information technology).

#### 3.2.5. Control Variables

Several demographic variables were added and controlled, including: the gender (0 = male, 1 = female); age (years); income (1 = below 2000 yuan (285 US$), 2 = 2001–4000 yuan (286–571 US$), 3 = 4001–6000 yuan (572–857 US$), 4 = 6001–8000 yuan (858–1142 US$), 5 = 8001–10,000 yuan (1143–1428 US$), 6 = over 10,000 yuan (1428 US$).

### 3.3. Analytic Strategy

SPSS 24.0 was first used to screen for multivariate outliers and to calculate the descriptive statistics for all study variables. Next, a Confirmatory Factor Analysis (CFA) was conducted in Mplus 8.0 to assess the discriminant validity and dimensionality of the focal constructs, including career calling, career loyalty, and Major-Occupation Match. Since the data were collected at the individual level, no hierarchical structure was involved, and thus the hypotheses were tested using Structural Equation Modeling (SEM).

The SEM analysis process was composed of four models. Model 1 examined the direct effect of career calling on career loyalty. Model 2 added the interaction term between career calling and average industry income to test the moderating effect of industry-level wage conditions on career loyalty. Model 3 tested the direct effect of career calling on Major-Occupation Match. Model 4 incorporated career loyalty into the model to examine the extent to which it mediates the relationship between career calling and Major-Occupation Match. The Akaike Information Criterion (AIC) value of each model was also presented for comparison, with lower AIC values indicating better model fit (Sutherland et al., 2023).

Second, the mediation effects (i.e., Hypothesis 2) were further examined using the SEM mediation command with bias-corrected bootstrap analysis (5000 resamples) and a 95% confidence interval (CI) through Mplus. If the CI did not include zero, the mediation effect was considered significant (Grant & Berry, 2011).

Finally, the moderated mediation effects (i.e., Hypothesis 4) were examined using SEM in Mplus by estimating the conditional indirect effects of career calling on Major-Occupation Match through career loyalty at different levels of average industry income. The significance of these effects was tested using the Monte Carlo method with a 95% CI.

## 4. Results

### 4.1. Preliminary Analysis

The results of the CFA were used to test the validity of the conceptual model. Two factors received the CFA examination, namely career calling and career loyalty. Specifically, the model fit was evaluated using χ^2^, root mean square error of approximation (RMSEA), standardized root mean square residual (SRMR), comparative fit index (CFI), and Tucker–Lewis index (TLI). As shown in Table 1, the hypothesized two-factor model demonstrated a superior fit compared to the single-factor model, with lower χ^2^/df, RMSEA, and SRMR values, and higher CFI and TLI indices. This provided evidence for the distinctiveness of career calling and career loyalty in the current sample of Chinese students.

In addition, the reliability and validity of the studied constructs were assessed using construct reliability (CR) and average variance extracted (AVE). As indicated in Table 2, all CR values exceeded the recommended threshold of 0.70, and all AVE values were greater than 0.60, confirming acceptable reliability and convergent validity for each construct.

Table 2 further presents the descriptive statistics, including means, standard deviations, and Pearson’s bivariate correlations among variables. The results show that career calling was positively associated with career loyalty (r = 0.376, *p* < 0.01), indicating that participants who perceived their work as a calling reported stronger loyalty to their career. Moreover, career loyalty was positively correlated with both average industry income (r = 0.211, *p* < 0.001) and Major-Occupation Match (r = 0.528, *p* < 0.001), suggesting that participants with higher loyalty were more likely to benefit from higher income levels and to be working in jobs aligned with their educational background. Career calling also showed significant positive correlations with industry income (r = 0.192, *p* < 0.001) and Major-Occupation Match (r = 0.288, *p* < 0.001). These findings provide initial evidence for the criterion-related validity of the developed measures.

### 4.2. Hypothesis Testing

Table 3 presents the results of the single-level SEM analysis across four models. More specifically, Table 3 shows that career calling was positively related to career loyalty (r = 0.316, *p* < 0.001; Model 1). As for the direct effect of career calling on Major-Occupation Match (Model 3), the effect was significant (r = 0.221, *p* < 0.001; Model 3), supporting Hypothesis 1.

Table 4 summarizes the mediation results. The indirect effect of career calling on Major-Occupation Match through career loyalty was significant (r = 0.131, *p* < 0.001; 95% CI [0.024, 0.238]), whereas the direct effect was nonsignificant (r = 0.032, ns). The total effect was significant (r = 0.163, *p* < 0.001; 95% CI [–0.078, 0.404]). These findings further confirm that career loyalty plays a complete mediating role in the relationship between career calling and Major-Occupation Match. More importantly, when career loyalty was included in the model (Model 4), the direct effect of career calling on Major-Occupation Match became nonsignificant (r = 0.032, ns; Model 4), whereas career loyalty itself showed a strong positive relationship with Major-Occupation Match (r = 0.327, *p* < 0.001; Model 4). This pattern suggests a full mediation effect of career loyalty, consistent with Hypothesis 2.

**Figure 2:**
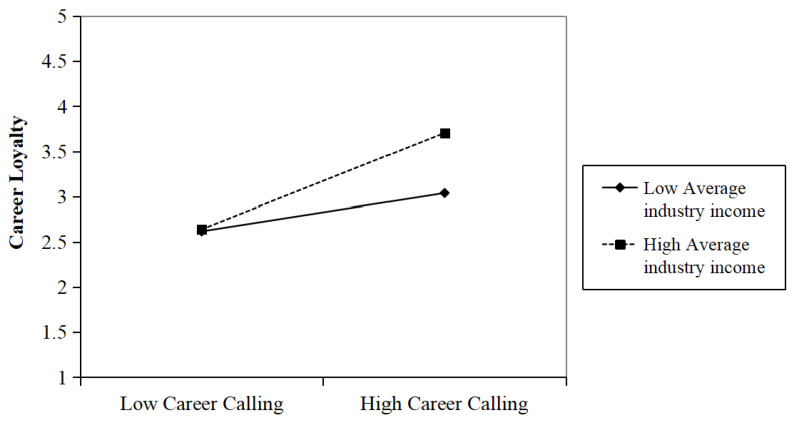
Simple slope of average industry income and graduate students’ career calling on their career loyalty.

When average industry income and its interaction with career calling were introduced (Model 2), the interaction term was significantly positive (r = 0.112, *p* < 0.001; Model 2), indicating that industry income strengthened the positive relationship between career calling and career loyalty. Table 5 presents the moderation effect of average industry income on the relationship between career calling and career loyalty. The moderation analysis revealed that when industry income was low (–1 SD), the effect of career calling on career loyalty was significant (r = 0.282, *p* < 0.001; 95% CI [0.142, 0.422]). When industry income was high (+1 SD), the effect was even stronger (r = 0.477, *p* < 0.001; 95% CI [0.291, 0.663]). The difference between high and low levels of industry income was significant (Δr = 0.195, *p* < 0.001; 95% CI [0.011, 0.379]), supporting Hypothesis 3 (Figure 2).

Finally, Table 6 presents the moderated mediation results. The average indirect effect of career calling on Major-Occupation Match through career loyalty, moderated by industry income, was significant (r = 0.201, *p* < 0.001; 95% CI [0.089, 0.313]). Under low industry income (–1 SD), the indirect effect was significant but weaker (r = 0.128, *p* < 0.001; 95% CI [0.063, 0.193]). Under high industry income (+1 SD), the indirect effect was much stronger (r = 0.274, *p* < 0.001; 95% CI [0.159, 0.389]). The difference between the two conditions was significant (Δr = 0.146, *p* < 0.001; 95% CI [0.043, 0.249]). These results provide strong support for Hypothesis 4, confirming that average industry income positively moderates the indirect relationship between career calling and Major-Occupation Match via career loyalty.

## 5. Discussion

### 5.1. Theoretical Implications

This study makes several theoretical contributions to the literature on career calling, person–environment fit, and career development. By drawing on a nationally representative sample of Chinese college graduates, the findings extend existing theories and provide new insights into the mechanisms and boundary conditions of career calling. Specifically, the study contributes to theory in at least four important ways.

#### 5.1.1. Extending the Theoretical Boundaries of Career Calling Beyond Helping Professions

Much of the extant research on career calling has been conducted in professions with strong prosocial orientations, such as medicine, nursing, and teaching (Zhao et al., 2022; Zhou et al., 2022). Within these occupational contexts, calling has been consistently linked to outcomes such as work engagement, job satisfaction, and reduced turnover intentions (Yoon et al., 2017; Yu et al., 2022). However, relatively little empirical work has examined whether the motivational power of calling extends to broader career contexts beyond traditionally “other-oriented” professions.

The present study contributes to this gap by demonstrating that career calling also plays a significant role in shaping career outcomes for general occupational groups, specifically among college graduates transitioning from higher education into the labor market. By analyzing a large and diverse national sample, the results indicate that calling exerts a robust influence on both job loyalty and the extent to which graduates pursue occupations aligned with their academic majors. This finding broadens the theoretical boundary of calling and highlights its explanatory power in more ordinary career domains that are not necessarily defined by altruistic missions.

From the perspective of person–environment (P–E) fit theory, this extension is theoretically significant because it illustrates that calling is not only salient when values of service and care are embedded in the occupational context, but also when individuals strive to align their professional identity, educational background, and personal values with their career paths (Xu et al., 2023). Thus, the study enriches our understanding of calling as a universal motivational construct, rather than one confined to public service occupations.

#### 5.1.2. Unveiling the Mediating Role of Job Loyalty in the Calling–Career Outcomes Relationship

Existing studies have primarily emphasized the direct effects of calling on work-related attitudes and behaviors, such as job involvement, satisfaction, and turnover intention (Duffy et al., 2018; J. Zhang & Han, 2023). In contrast, the current research advances the literature by identifying job loyalty as a key mediating mechanism through which calling influences Major-Occupation Match. The findings show that graduates with stronger callings are more loyal to their chosen career paths, and this loyalty subsequently increases the likelihood of pursuing jobs consistent with their academic majors.

This mechanism resonates strongly with the Conservation of Resources (COR) theory (Nemțeanu et al., 2022). COR theory posits that individuals strive to acquire, retain, and protect valued resources. Calling can be understood as an important psychological resource that provides meaning and resilience in the face of career challenges (Jin et al., 2023). However, the effect of calling is not immediate; rather, it is channeled through enhanced job loyalty, which represents a resource investment strategy. By committing to a career path, individuals consolidate and protect their psychological resource of calling, which in turn facilitates resource gains such as professional growth and person–job congruence.

This insight contributes to the broader theoretical conversation by highlighting that calling functions less as a direct driver of behavioral outcomes and more as a resource catalyst that strengthens individuals’ psychological commitment to their profession. In doing so, the study adds nuance to our understanding of how motivational resources are translated into concrete career decisions through intermediate attitudinal processes.

#### 5.1.3. Introducing Industry Income Level as a Boundary Condition for Calling Effects

A third contribution lies in incorporating industry-level income as a contextual moderator of the calling–loyalty relationship. Prior research on calling has predominantly emphasized its intrinsic, value-driven nature, often underplaying the role of economic rewards and structural conditions (Swen, 2020; Ulfa et al., 2021). The findings of this study suggest that although calling is an internal motivational force, its effects are embedded within broader socioeconomic contexts. Specifically, graduates working in higher-paying industries exhibited a stronger linkage between calling and job loyalty, whereas this relationship was weaker in lower-paying sectors.

This moderation effect advances theoretical understanding in at least two ways. First, from the perspective of COR theory, industry income represents a form of contextual resource availability that can either amplify or constrain the ability of individuals to invest and sustain their calling (Bardoel & Drago, 2021). In high-income industries, individuals are able to accumulate tangible resources (e.g., financial rewards, social recognition), which in turn reinforce the motivational resource of calling. Conversely, in low-income contexts, the absence of such external reinforcements may lead to resource depletion, weakening the impact of calling on loyalty.

Second, through the lens of P–E fit theory, the findings suggest that fit is not determined solely by the alignment of personal values and job characteristics but also by the congruence between individual motivations and external reward structures (Lee et al., 2021). When calling is reinforced by attractive industry-level income, the sense of fit is magnified, thereby strengthening loyalty. This contextualized perspective broadens P–E fit theory by integrating economic structures into the motivational alignment process.

#### 5.1.4. Bridging the Literature on Career Calling and Major-Occupation Match

Finally, the study integrates the career calling literature with the domain of Major-Occupation Match, which has been extensively studied in education economics and labor market research (Robertson et al., 2024). Most existing studies have explained Major-Occupation Match in terms of human capital, skill utilization, and labor market demand. This perspective, while valuable, often neglects the role of psychological factors and intrinsic motivations.

By demonstrating that calling significantly predicts whether graduates enter jobs aligned with their field of study, the current study underscores that professional congruence is not solely a product of market mechanisms but also of motivational resources and value orientations. In particular, the mediating role of job loyalty highlights that alignment between education and employment reflects a psychological process of commitment and persistence rather than merely economic rationality.

This contribution enriches both bodies of literature by introducing a cross-disciplinary perspective: Major-Occupation Match is simultaneously an economic allocation issue and a psychological fit issue. From the standpoint of P–E fit theory, calling strengthens individuals’ drive to seek alignment between their educational preparation and occupational choice, thereby enhancing both subjective career satisfaction and objective labor market efficiency.

### 5.2. Summary of Theoretical Contributions

Taken together, these four contributions deepen our theoretical understanding of career calling in several ways. First, they establish calling as a motivational construct applicable across diverse occupations, extending its theoretical scope beyond helping professions. Second, they identify job loyalty as a crucial mediating mechanism, advancing knowledge of the psychological processes through which calling influences career outcomes. Third, they reveal the contextual role of industry income, integrating socioeconomic conditions into COR and P–E fit perspectives. Fourth, they bridge calling research with Major-Occupation Match studies, offering a more comprehensive account of how individual motivations and structural contexts jointly shape career development.

By situating these insights within the frameworks of COR theory and P–E fit theory, the study not only enriches both theoretical traditions but also provides a more holistic understanding of how internal resources and external environments interact to shape career trajectories.

#### 5.2.1. Practical Implications

This study also provides several practical implications at the levels of education policy, organizational management, socio-economic development, and individual career planning. Together, these implications highlight the importance of fostering career calling, enhancing job loyalty, and considering contextual economic conditions in shaping graduates’ career outcomes.

#### 5.2.2. Implications for Higher Education and Policymakers

The finding that career calling significantly promotes Major-Occupation Match underscores the importance of embedding values-oriented guidance into higher education. Universities often emphasize technical competence and employability but tend to neglect the cultivation of broader mission-driven attitudes. By integrating courses on professional mission, civic responsibility, and value-based career planning into the curriculum, higher education institutions can help students better align their academic interests with social needs (Duffy et al., 2018). Furthermore, alumni mentorship and role-modeling initiatives can provide tangible examples of graduates who successfully integrate their professional calling with career development (Ronkainen et al., 2022). For policymakers, this suggests that career education policies should not be limited to labor market matching mechanisms but should also emphasize the cultivation of intrinsic motivation and professional identity, thereby supporting sustainable employability and reducing skill–job mismatches (Zhou et al., 2022).

#### 5.2.3. Implications for Organizations and Employers

The mediating role of job loyalty suggests that organizations should not only assess candidates’ technical competencies but also attend to their value alignment and sense of calling. Employees with a strong calling are more likely to develop loyalty to their organizations, which in turn promotes greater professional stability and alignment with their field of study (Rosewell & Ashwin, 2019). To leverage this dynamic, employers can foster employee loyalty through clear career development pathways, supportive leadership practices, and organizational cultures that emphasize purpose and contribution rather than merely transactional rewards (Kim & Kiura, 2023). Value-based recruitment, onboarding processes that highlight organizational mission, and recognition systems for contributions beyond formal role expectations can also reinforce employees’ commitment. For industries with high turnover rates, strengthening organizational cultures that support calling and loyalty can be a cost-effective way to retain talent and enhance professional match outcomes.

#### 5.2.4. Implications for Socio-Economic Policy and Labor Market Reform

The moderating effect of industry income reveals that career calling is embedded in broader socio-economic contexts. While calling is an intrinsic motivation, its effects are more pronounced when coupled with favorable external conditions, such as adequate compensation. This highlights that improving industry-wide remuneration is not only an economic necessity but also a means to reinforce the realization of individuals’ professional calling (Zheng, 2024). From a policy perspective, governments could design wage policies, subsidies, or incentive structures to increase the attractiveness of industries critical to national development but traditionally underpaid, such as public health, education, or social services (Khatib, 2024). By improving pay equity across sectors, policymakers can promote a virtuous cycle where intrinsic motivation and external rewards are mutually reinforcing, thereby reducing talent drain from essential but low-paying industries. Moreover, this finding resonates with the Person–Environment (P–E) fit framework, which emphasizes that congruence between personal values and external conditions determines long-term career satisfaction and retention (Kivrak et al., 2025).

#### 5.2.5. Implications for Individual Career Development

At the individual level, the findings highlight the importance of considering both intrinsic calling and extrinsic factors, such as compensation, in career decision-making. Graduates who experience a strong sense of calling are more likely to remain loyal to their profession, even in the face of challenges. However, this loyalty is strengthened when external rewards are adequate, suggesting that individuals should strategically evaluate both their intrinsic motivations and the external conditions of their chosen industry. For individuals, stronger career loyalty can foster a sense of meaning, stability, and long-term skill accumulation, which in turn may support career growth, so employees should cultivate career loyalty in a balanced way (Veloso et al., 2021; Wijonarko et al., 2024). Career counselors and advisors can play a crucial role in helping students and early-career employees weigh these dimensions, encouraging them to pursue paths that balance mission-driven fulfillment with sustainable livelihood. This dual consideration can reduce premature turnover and ensure that individuals build careers that are both meaningful and economically viable (Wrzesniewski et al., 1997).

### 5.3. Summary of Practical Value

Overall, the practical contributions of this study can be summarized as follows: universities and policymakers should embed value-based education to cultivate calling, organizations should strengthen loyalty through supportive practices, governments should improve wage structures to align intrinsic and extrinsic motivations, and individuals should consider both mission and material conditions when planning careers. These implications collectively emphasize that the realization of career calling is not merely a personal psychological process but one shaped by multi-level interactions across educational, organizational, and socio-economic systems.

## 6. Limitations

Despite the contributions of this study, several limitations should be acknowledged, which also provide directions for future research. First, the data were collected from a single national context, focusing on Chinese graduates. While this setting is highly relevant given the rapid expansion of higher education and the increasing concern with graduate employability in China, it may limit the generalizability of the findings to other cultural or institutional contexts. Cross-national comparative studies could provide stronger evidence for the universality or cultural specificity of the relationships between career calling, job loyalty, and Major-Occupation Match.

Second, the measurement of industry-level income was based on aggregated statistical indicators rather than individual perceptions. While this macro-level approach captures objective differences across sectors, it may not fully reflect employees’ subjective experiences of compensation fairness or sufficiency, which are also critical in shaping their career outcomes. Subsequent studies could integrate both objective economic indicators and subjective pay perceptions to provide a more nuanced understanding of the moderating role of industry income.

Third, the study examined only a limited set of mediating and moderating mechanisms. Career development is a complex process shaped by multiple individual and contextual factors, including personality traits, leadership styles, labor market dynamics, and institutional policies. While job loyalty and industry income are important explanatory variables, future research could extend the model by incorporating other mediators, such as organizational commitment or work engagement, and other moderators, such as leadership support or job autonomy. Expanding the scope of inquiry will deepen the theoretical understanding of how career calling influences professional outcomes in diverse organizational and societal contexts.

Finally, while our use of industry-level categories provides a practical proxy for analyzing career outcomes, it also reflects a form of “lumping” rather than “splitting” of occupations. This approach constrains the extent to which we can speak to more nuanced, individual-level employment trajectories. For instance, the data do not allow us to determine whether graduates are employed within the occupational category aligned with their field of study, or whether they have entered a different industry where their skills are transferable. As a result, our findings may obscure meaningful variation in career progression and the alignment between education and employment. In addition, the operationalization of Major-Occupation Match as a binary distinction between “matched” and “mismatched” outcomes represents an analytical simplification. While this approach is consistent with prior research and reflects the structure of the available data, it does not fully capture the complexity of labor market realities. By collapsing these diverse trajectories into a binary categorization, our analysis may obscure meaningful variation in the degree of alignment between education and employment. Future research could address this limitation by developing more fine-grained measures of match, such as ordinal categories (e.g., fully matched, partially matched, mismatched) or continuous indices of fit, which would enhance the validity and interpretive power of findings on the education–employment nexus.

For future research, it would be valuable to pursue cross-cultural comparisons to assess the extent to which the observed dynamics are culturally specific or generalizable. In addition, future studies should incorporate factors such as work engagement, career adaptability, or social support to develop a more nuanced and multidimensional understanding of how calling translates into labor market outcomes. Furthermore, the present study is limited by sample attrition. Future research should address this issue by conducting systematic attrition analyses, applying appropriate weighting techniques, and strengthening follow-up procedures in order to mitigate potential biases in representativeness.

In sum, while these limitations do not undermine the validity of the present findings, they suggest promising avenues for future research. Addressing them will help build a more comprehensive and context-sensitive understanding of career calling and its implications for graduates’ career trajectories.

## 7. Conclusions

This study advances the understanding of career calling in the context of graduate employment by examining its impact on Major-Occupation Match and the mediating role of job loyalty. Drawing on Conservation of Resources (COR) theory and person–environment (P–E) fit theory, the findings demonstrate that calling serves as an important personal resource that not only motivates individuals to align their career choices with their academic training but also fosters stronger loyalty toward their occupations. Job loyalty, in turn, emerged as a crucial psychological mechanism that translates calling into tangible employment outcomes. Moreover, the moderating effect of industry income highlights the importance of structural and economic conditions, suggesting that higher industry-level compensation enhances the positive influence of calling on career alignment.

The results underscore the need to integrate both personal and contextual perspectives when analyzing career development. By showing that calling interacts with organizational loyalty and broader economic structures, this research contributes to a more holistic understanding of how graduates navigate the transition from education to employment. Practically, the findings offer insights for educators, employers, and policymakers aiming to foster career sustainability, talent retention, and effective human capital utilization. Taken together, the study highlights the significance of creating environments in which individual aspirations, organizational values, and economic incentives are mutually reinforcing, thereby enabling young professionals to realize their career calling and achieve long-term success.

## Figures and Tables

**Table 1:** Results of confirmed factor analysis.

Model	Factor	χ^2^	df	χ^2^/df	RMSEA [95% CI]	SRMR	CFI	TLI
Two-Factor Model	CC, CL	342.61	76	4.50	0.070 [0.062; 0.078]	0.013	0.907	0.913
Single-Factor Model	CC + CL	415.27	80	5.19	0.083 [0.071; 0.095]	0.018	0.833	0.872

CC = Career Calling, CL = Career Loyalty.

**Table 2:** Means, standard deviations, reliability, and Pearson’s bivariate correlations among variables.

Variables	Mean	SD	CR	AVE	1	2	3	4
Individual Level (n = 2025)								
1. Career Calling	4.07	0.54	0.80	0.71	—			
2. Career Loyalty	4.04	0.46	0.74	0.72	0.376 **	—		
3. Average Industry Income (Unit: 10 thousand Yuan)	9.7730	4.5332	0.78	0.72	0.192 ***	0.211 ***	—	
4. Major-Occupation Match	0.554	0.497	0.79	0.73	0.288 **	0.528 **	0.186 **	—

** *p* < 0.01; *** *p* < 0.001.

**Table 3:** Results of SEM Modeling.

Variable	Career Loyalty		Major-Occupation Match	
	Model 1	Model 2	Model 3	Model 4
Career Calling	0.316 (0.162) ***	0.136 (0.151) *	0.221 (0.131) ***	0.032 (0.102)
Career Loyalty				0.327 (0.167) ***
Average Industry Income		0.184 (0.102) ***		
Average Industry Income × Career Calling		0.112 (0.122) ***		
R^2^	0.571	0.753	0.485	0.652
AIC	3132.193	2231.207	2205.246	1717.219

* *p* < 0.05; *** *p* < 0.001. Values in the parentheses are the SE value for each variable.

**Table 4:** The summary result of the Mediation Effect.

Condition				Mediation Effects		
Mediation Effects of Career Calling on Major-Occupation Match via Career Loyalty						
				Career Calling		
				Coefficient	LL 95% CI	UL 95% CI
Direct effect				0.032 (0.142)	−0.102	0.166
Indirect effect				0.131 (0.126) ***	0.024	0.238
Total effect				0.163 (0.112) ***	−0.078	0.404

*** *p* < 0.001. Values in the parenthesis are SE value for each variable.

**Table 5:** The summary result of the Moderation Effect.

Conditions	Indirect Effects		
Moderation Effects of Average Industry Income on the Relationship Between Career Calling and Career Loyalty			
	Career Loyalty		
	Coefficient	LL 95% CI	UL 95% CI
Low (1 − SD)	0.282 (0.132) ***	0.142	0.422
High (1 + SD)	0.477 (0.212) ***	0.291	0.663
Difference	0.195 (0.107) ***	0.011	0.379

*** *p* < 0.001. Values in the parentheses are the SE value for each variable.

**Table 6:** Summary of the Indirect Effects of the moderated mediation Model.

Conditions	Indirect Effects		
Moderation Effects of Average Industry Income on the Relationship Between Career Calling and Major-Occupation Match Via Career Loyalty			
	Receive Rewards		
	Coefficient	LL 95% CI	UL 95% CI
Average Indirect Effect	0.201 (0.182) ***	0.089	0.313
Low (1 − SD)	0.128 (0.141) ***	0.063	0.193
High (1 + SD)	0.274 (0.173) ***	0.159	0.389
Difference	0.146 (0.109) ***	0.043	0.249

*** *p* < 0.001. Values in the parentheses are the SE value for each variable.

## Data Availability

The datasets presented in this article are not readily available because of the ethical limitation. Due to the nature of this research, participants of this study did not agree for their data to be shared publicly, so supporting data is not available.

## Appendix A

**Table A1:** Industry Categories in Urban Non-private and Private Units.

Urban Non-Private Units	Urban Private Units
Agriculture, Forestry, Animal Husbandry, and Fishery	Agriculture, Forestry, Animal Husbandry, and Fishery
Mining	Mining
Manufacturing	Manufacturing
Production and Supply of Electricity, Heat, Gas, and Water	Production and Supply of Electricity, Heat, Gas, and Water
Construction	Construction
Wholesale and Retail Trade	Wholesale and Retail Trade
Transportation, Storage, and Postal Services	Transportation, Storage, and Postal Services
Accommodation and Catering Services	Accommodation and Catering Services
Information Transmission, Software, and Information Technology Services	Information Transmission, Software, and Information Technology Services
Financial Industry	Financial Industry
Real Estate	Real Estate
Leasing and Business Services	Leasing and Business Services
Scientific Research and Technical Services	Scientific Research and Technical Services
Water Conservancy, Environment, and Public Facilities Management	Water Conservancy, Environment, and Public Facilities Management
Resident Services, Repair, and Other Services	Resident Services, Repair, and Other Services
Education	Education
Health and Social Work	Health and Social Work
Culture, Sports, and Entertainment	Culture, Sports, and Entertainment
Public Administration, Social Security, and Social Organizations	—
